# Pyoderma Gangrenosum: A Presenting Feature of Rheumatoid Arthritis

**DOI:** 10.7759/cureus.64288

**Published:** 2024-07-10

**Authors:** Pooja Chaurasia, Nishtha Malik, Shubham Deokar, Kirti S Deo, Aayush Gupta

**Affiliations:** 1 Dermatology, Dr. D. Y. Patil Medical College, Hospital and Research Centre, Dr. D. Y. Patil Vidyapeeth, Pune (Deemed to Be University), Pune, IND; 2 Dermatology, Venereology and Leprosy, Dr. D. Y. Patil Medical College, Hospital and Research Centre, Dr. D. Y. Patil Vidyapeeth, Pune (Deemed to Be University), Pune, IND

**Keywords:** rheumatoid arthritis, dermato-rheumatology, neutrophilic dermatosis, association, pyoderma gangrenosum (pg)

## Abstract

Pyoderma gangrenosum (PG) is an uncommon inflammatory disorder that exhibits a range of clinical manifestations and levels of severity. It frequently occurs alongside an underlying condition, most often inflammatory bowel disease. PG, Sweet syndrome, palisaded neutrophilic granulomatous dermatitis (PNGD), interstitial granulomatous dermatitis (IGD) and rheumatoid neutrophilic dermatitis may be associated with rheumatoid arthritis (RA).

We present a case of a 65-year-old woman with disseminated dermatosis to the hands, abdomen, buttocks, and lower limbs. The dermatosis presented with numerous ulcers of varying shapes, featuring clean bases, undermined edges, and a purplish erythematous appearance. Further investigations, including imaging studies and RA factor and anti-cyclic citrullinated peptide (anti-CCP) levels, led us to the diagnosis of RA.

This case indicates that RA may be frequently undiagnosed and untreated in other patients with PG, as ulcers on the lower extremities can often be the main reason for seeking medical attention.

## Introduction

Neutrophilic dermatosis (ND) encompasses a range of clinical conditions characterized by the presence of mature polymorphonuclear leukocytes at various levels within the epidermis, dermis, and subcutis upon histological examination. Within this group, pyoderma gangrenosum (PG), Sweets syndrome, and erythema elevatum diutinum (EED) are well-defined entities that can be associated with rheumatoid arthritis (RA) [1].

PG can occur on its own; however, it is more usually associated with an underlying illness. Up to 75% of PG patients are related to inflammatory arthritis, inflammatory bowel illnesses, or hematological cancers [2]. Proposed causes of this ulcerating skin condition include hereditary, autoimmune, and autoinflammatory processes, which also play a role in the pathophysiology of RA.

## Case presentation

**Figure 1:**
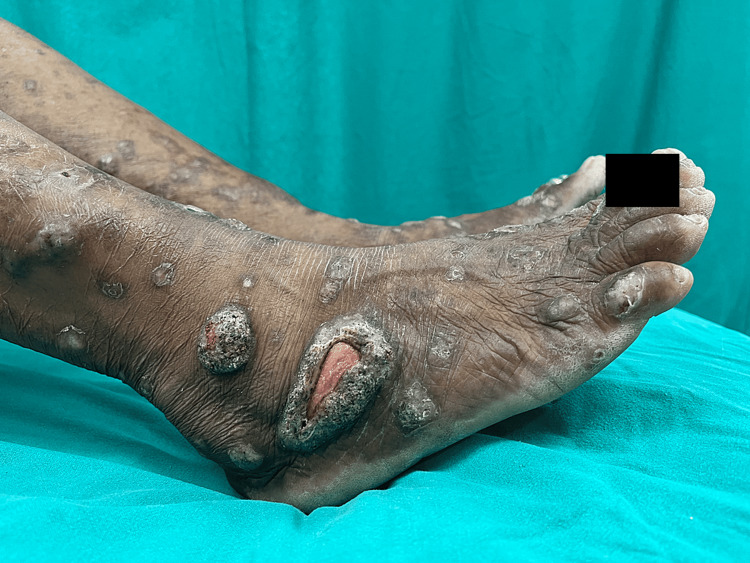
Cutaneous examination findings Multiple hyperkeratotic plaques are present on the lower limbs, along with an ulcer presenting an erythematous base.

A 65-year-old female, with a known case of RA for four years, presented with an insidious onset of painful, itchy lesions, over her lower extremities, buttocks, and abdomen over a period of two years. She reported a rapid evolution of the lesions that resulted in the formation of multiple well-defined ulcers of varying stages, having an erythematous base, an undermined edge, and a raised hyperkeratotic border (Figure 1).

**Figure 2:**
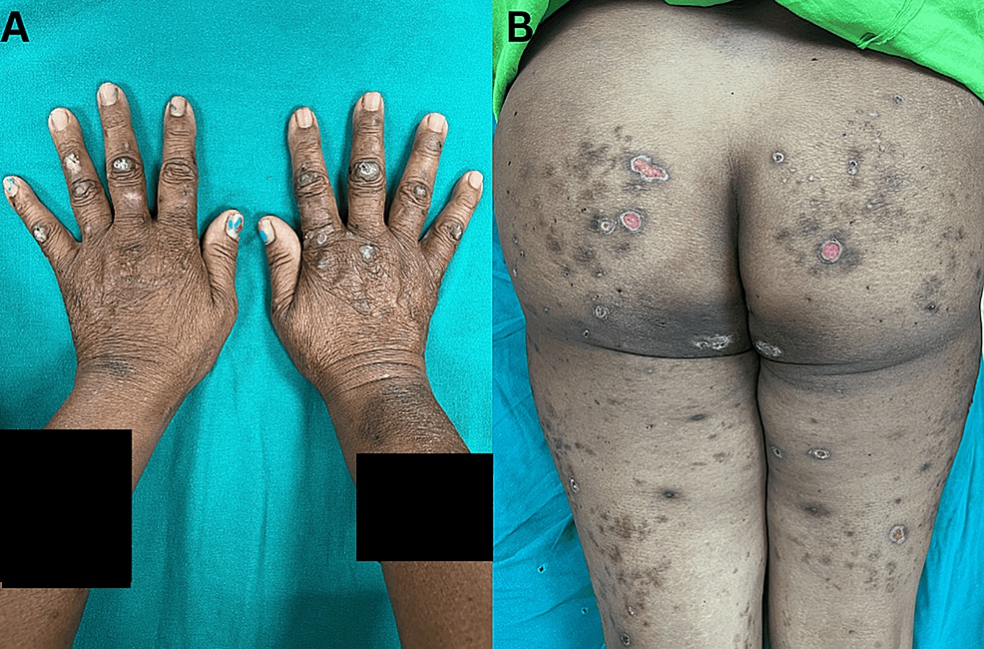
Lesions depicting the extent of involvement (A) Hyperpigmented and hyperkeratotic plaques with crusting over bilateral metacarpophalangeal joints; (B) Multiple ulcers having an erythematous base, an undermined edge, and a violaceous hue.

Multiple hyperpigmented and hyperkeratotic plaques with crusting were present over bilateral metacarpophalangeal joints, arms, legs, and feet (Figures 2A, 2B).

Her history included irregular treatment with non-steroidal anti-inflammatory drugs (NSAIDs), without disease-modifying antirheumatic drugs (DMARDs), resulting in worsening of morning stiffness and joint pain. Laboratory investigations revealed anti-cyclic citrullinated peptide (anti-CCP) as 78.5 (N>5) and RA factor as 431 (N>20). C-reactive protein was raised to 40 mg/L (reference: <5mg/L); and the erythrocyte sedimentation rate was elevated at 46 mm/h (reference: <30 mm/h after one hour) while complete blood count was within the normal range.

**Figure 3:**
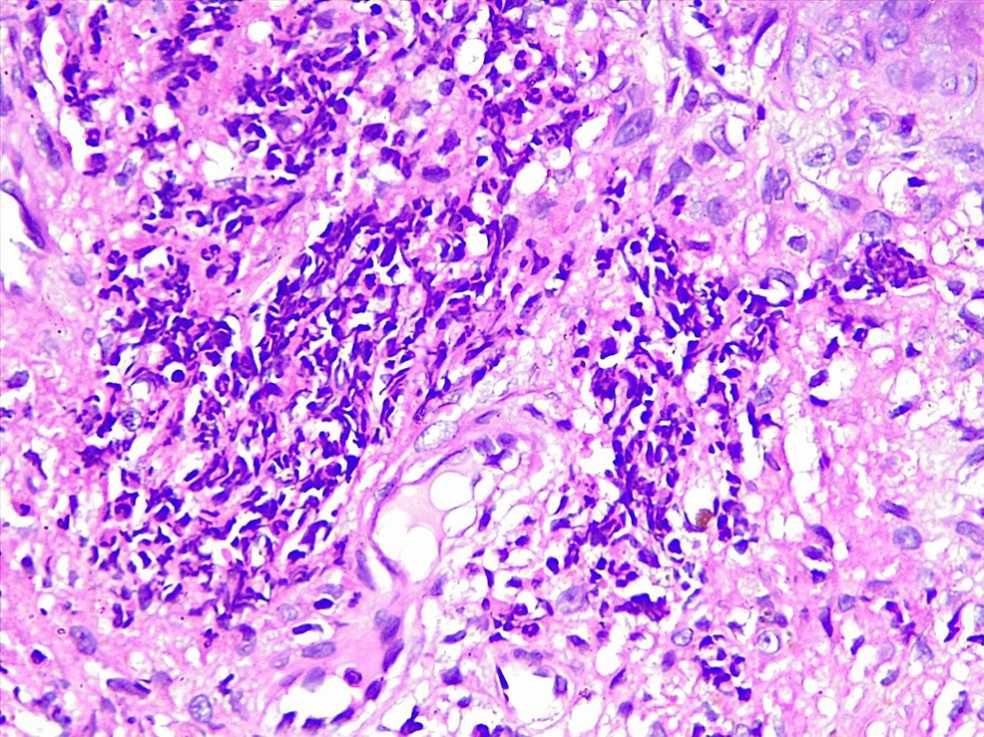
Histopathological findings The inflammatory infiltrate is predominantly composed of neutrophils in the epidermis and marked papillary edema (H&E, 40X).

Histopathology showed an inflammatory infiltrate predominantly composed of neutrophils in the epidermis, marked papillary edema, and dense neutrophilic infiltrate in the dermis, confirming a diagnosis of PG (Figure 3).

She was primarily treated with 30 mg of prednisolone daily to which she had a marked improvement of the lesions. Along with this, a weekly dose of 15 mg of methotrexate and 200 mg of hydroxychloroquine was initiated, with topical application of clobetasol ointment.

## Discussion

Brunstinge O'Larry originally described PG more than 70 years ago [3]. This is a rare, non-infectious, chronic, relapsing inflammatory dermatosis that causes local damage and is often related to other disorders. Although more frequent in adults, it can also afflict children on rare occasions. The most affected age range is 25-55 years old, with women having a higher prevalence of the condition. PG is often associated with various other conditions, including seronegative arthritis, ankylosing spondylitis, monoclonal gammopathy, myelodysplasia, PASH syndrome (pyogenic sterile arthritis, PG, and acne syndrome), PAPA syndrome (pyogenic arthritis, PG, and acne syndrome) and PA-PASH syndrome (pyogenic arthritis, PG, acne and suppurative hidradenitis syndrome) [4].

The pathogenesis of PG is intricate and involves a significant disruption of both innate and adaptive immune components in persons who are genetically susceptible. The follicular unit is widely acknowledged as the likely initial target [5]. Neutrophil dysfunction, namely chemotaxis abnormalities, may contribute to PG [6]. The presence of T helper 17/T helper 1-skewed inflammation and excessive inflammasome activation results in an imbalanced environment dominated by neutrophils, with elevated levels of tumor necrosis factor (TNF)-α, interleukin (IL)-1α, IL-8, IL-1β, IL-12, IL-15, IL-17, and IL-23 [5].

There are four distinct clinical manifestations of PG. The most common is the ulcerative variant, typically associated with inflammatory bowel disease (IBD) and RA. The pustular form linked to IBD is characterized by painful pustules with an erythematous halo [7]. The bullous type, associated with myeloproliferative diseases, features superficial hemorrhagic blisters and scarring. The vegetative form presents as a superficial, painless, single ulcer that is not associated with systemic disease [8]. Histologically, PG lesions are nonspecific, showing lymphocytic vasculitis along the erythematous margin and polymorphonuclear abscesses. Diagnosis is usually made through clinical exclusion.

RA is a systemic autoimmune inflammatory disease characterized by persistent, symmetric, and erosive polyarthritis, particularly in smaller joints. RA can cause extra-articular symptoms such as skin ulcers, rheumatoid nodules, vasculitis, and necrotic lesions of PG. Our patient presented with acquired widespread ulcerated skin lesions characteristic of PG, along with a history of pain in the small joints and morning stiffness, confirmed on laboratory evaluation as RA.

PG and rheumatic diseases are frequently associated, sometimes as high as 37% [9]. Both share a common T cell abnormality responsible for the destruction of pilosebaceous units in PG. Ulcerative PG having a predilection for the lower limbs closely mimics rheumatoid vasculitis or leg ulcers due to predisposing factors such as skin fragility, arterial disease, and venous insufficiency [10]. Thus, inspection of the skin would be a useful diagnostic tool in patients with RA, particularly in determining diagnosis and planning treatment. The treatment of PG is challenging due to the absence of a gold-standard therapy and the lack of randomized controlled trials. The treatment approach is primarily based on the clinician’s experience, as there are no established therapeutic guidelines. Given the immune system abnormalities underlying the disease, immunosuppressive therapy forms the cornerstone of treatment, which varies according to the severity and extent of the condition [11].

Due to their tenderness, PG lesions require wound care, dressings, compression, and pain management. Immunosuppressive medication can be topically or systemically applied depending on illness severity. Corticosteroids and tacrolimus are topical therapies, although intralesional injections may aggravate pathergy [12]. Systemic immunosuppression is needed for severe instances. Oral corticosteroids (0.5-1 mg/kg/day) and high-dose pulsed methylprednisolone (1 g/day for five days) can quickly retreat ulcers and relieve pain, although they can cause osteoporosis, adrenal suppression, and infection risk. Cyclosporine (alone or with corticosteroids), dapsone, methotrexate, azathioprine, colchicine, mycophenolate mofetil, sulfasalazine, thalidomide, and cyclophosphamide are other systemic treatments [13].

PG lesions heal faster with anti-TNF-α inhibitors like infliximab, etanercept, and adalimumab than steroids alone [14]. Various cytokine-targeting therapies exist, such as IL-17 (secukinumab), IL-12/IL-23 (ustekinumab), IL-1β (canakinumab), IL-1 receptor I (anakinra), JAK (tofacitinib, ruxolitinib), IL-6 (tocilizumab) and phosphodiesterase 4 (apremilast). Intravenous immunoglobulin (IVIG) has also been effective as an adjuvant treatment [15].

Despite advances in RA treatment, the incidence of extra-articular symptoms has remained consistent throughout time. PG patients with RA are more resistant to therapy than those without arthritis [9], as they constitute a challenging subgroup with ulcers likely stemming from distinctive underlying pathophysiological factors.

## Conclusions

The presence of multiple autoimmune conditions in patients like the one described in this context underscores the importance of ongoing monitoring for the development of new autoimmune diseases. Both rheumatologists and dermatologists should have a comprehensive understanding of the associations of RA to facilitate a coordinated therapeutic approach for addressing both cutaneous and extra-cutaneous afflictions.
